# Measuring everyday functional competence using the Rasch assessment of everyday activity limitations (REAL) item bank

**DOI:** 10.1007/s11136-017-1627-0

**Published:** 2017-06-21

**Authors:** Martijn A. H. Oude Voshaar, Peter M. ten Klooster, Harald E. Vonkeman, Mart A. F. J. van de Laar

**Affiliations:** 1 Arthritis Center Twente, Department of Psychology, Health and Technology, University of Twente, PO Box 217, 7500 AE Enschede, The Netherlands; 2 Arthritis Center Twente, Department of Rheumatology and Clinical Immunology, Medical Spectrum Twente, Enschede, The Netherlands

**Keywords:** Item response theory, Item bank, Computerized adaptive testing, Physical function, Activity limitations, Rasch

## Abstract

**Objective:**

Traditional patient-reported physical function instruments often poorly differentiate patients with mild-to-moderate disability. We describe the development and psychometric evaluation of a generic item bank for measuring everyday activity limitations in outpatient populations.

**Study design and setting:**

Seventy-two items generated from patient interviews and mapped to the International Classification of Functioning, Disability and Health (ICF) domestic life chapter were administered to 1128 adults representative of the Dutch population. The partial credit model was fitted to the item responses and evaluated with respect to its assumptions, model fit, and differential item functioning (DIF). Measurement performance of a computerized adaptive testing (CAT) algorithm was compared with the SF-36 physical functioning scale (PF-10).

**Results:**

A final bank of 41 items was developed. All items demonstrated acceptable fit to the partial credit model and measurement invariance across age, sex, and educational level. Five- and ten-item CAT simulations were shown to have high measurement precision, which exceeded that of SF-36 physical functioning scale across the physical function continuum. Floor effects were absent for a 10-item empirical CAT simulation, and ceiling effects were low (13.5%) compared with SF-36 physical functioning (38.1%). CAT also discriminated better than SF-36 physical functioning between age groups, number of chronic conditions, and respondents with or without rheumatic conditions.

**Conclusion:**

The Rasch assessment of everyday activity limitations (REAL) item bank will hopefully prove a useful instrument for assessing everyday activity limitations. T-scores obtained using derived measures can be used to benchmark physical function outcomes against the general Dutch adult population.

**Electronic supplementary material:**

The online version of this article (doi:10.1007/s11136-017-1627-0) contains supplementary material, which is available to authorized users.

## Introduction

Physical function can be defined as a person’s ability to engage in activities that require physical movement and exertion with the purpose of performing everyday tasks or needs. It is a central component of health-related quality of life and a key outcome in its own right across many medical conditions [1–7]. The level of self-reported physical disability experienced by individuals has been shown to vary widely within conditions for which physical function is commonly assessed [8, 9]. A fixed-length questionnaire that includes items relevant to each of these levels would have to include many questions that would not all be relevant for individual respondents. Since short questionnaires are usually preferred for feasibility reasons, it has proven challenging to develop fixed-length questionnaires that adequately measure the variety of physical function levels that might occur within a population of interest [10–12].

Item response theory (IRT)-based item banking has been promoted as a powerful solution for overcoming well-known limitations of fixed-length instruments, such as floor and ceiling effects or insensitivity to change [13–15]. IRT is a framework for modeling item response data in which items and respondents are located on a common scale [16, 17]. Once the scale location of a set of items has been estimated precisely, any number and combination of items in the resulting item bank can be used to estimate the scale location of future respondents from the same population. Computerized adaptive testing (CAT) is an application of IRT that utilizes this feature to optimize measurement precision of individual assessments and reduce the number of items that need to be administered for a precise estimate, in which an algorithm selects items that best match the individual’s estimated level of the measured trait in real-time [18]. This could yield shorter, potentially equiprecise assessments, depending on the characteristics of the item bank.

However, studies in outpatient settings have shown currently available physical function item banks to poorly differentiate patients with mild-to-moderate disability [19–21] and most items to target lower levels of function [22–26]. This likely reflects that much of their content is derived from previously validated questionnaires [23, 27]. Many such questionnaires were developed for use in populations with severe disabilities and focus on basic activities of daily living (ADL) that are essential for independent self-care such as toileting, grooming, and eating, as well as basic mobility functions such as getting in and out of bed [28–30].

Since the precision with which scores can be estimated is optimal if an instrument’s items are well matched with trait levels of the respondents, a balanced coverage of the full spectrum of physical function would likely further improve measurement performance of physical function item bank applications in a variety of settings. Previous studies suggest that items reflecting various activities that people routinely engage in as part of managing their domestic responsibilities better match the levels of disability typically experienced by outpatients compared with the basic ADL. [31–37]. However, item content analyses have shown that such activities are infrequently included in the validated instruments that are now in widespread use [38–43]. The objective of the present work was to develop a new item bank that assesses disability in complex activities typically encountered in the daily lives of independently living individuals and to provide an initial evaluation of its measurement properties using an empirical CAT simulation.

## Methods

### Item pool development

The items were designed to capture the health concepts included in the International Classification of Functioning, Disability and Health (ICF), domestic life domain, using content that was derived as much as possible from people with first-hand experience with physical disability. An initial list of activities was derived from the responses obtained in a survey among a convenience sample of 103 consecutive patients attending a rheumatology outpatient clinic in the Netherlands. Characteristics of these patients are summarized in supplementary Table 1. Patients were asked: “Many people with [medical condition] experience difficulties in performing daily physical activities, such as climbing stairs or gardening. When you think about your [medical condition], what limitations in daily activities bother or upset you the most?” We linked the activities mentioned by patients to the ICF [44] and selected the 143 (39%) activities that were mapped to any third-level code within the ICF domestic life chapter. The process of binning and winnowing, which involves grouping together related concepts and deleting redundant content from these groups [27], was applied to these 143 activities, yielding a list of 57 sufficiently distinct activities. The degree to which this list comprehensively covered all ICF domestic life second-level codes was reviewed and 15 additional items were written by the project team to ensure a balanced coverage of the ICF domestic life second-level codes. We used slightly adapted versions of the PROMIS physical function item bank and health assessment questionnaire disability index (HAQ) item stems (“are you physically able to [activity]?”) and response options (without any difficulty/with a little difficulty/with some difficulty/with a lot of difficulty/cannot do), since these have been extensively tested in qualitative research and are widely used [27, 30].

**Table 1:** Sample characteristics

Age, years mean (SD) min–max	50.36 (17.99) 16–97
Sex, *n* (%)	
Male	524 (46.5%)
Female	604 (53.5%)
Educational level, *n* (%)^a^	
Low	314 (28%)
Middle	416 (37%)
High	394 (35%)
Occupational status, *n* (%)	
Remuneratively employed	546 (48.4%)
Pensioned	261 (23.1%)
Student	107 (9.5%)
Housekeeper	85 (7.5%)
Looking for work	54 (4.7%)
Unable to work	41 (3.6%)
Voluntarily employed	29 (2.6%)
Other	5 (0.4%)
Self-reported diagnosis of chronic condition, *n* (%)	
Osteoarthritis	162 (14.4%)
Diabetes mellitus	65 (5.8%)
Asthma	65 (5.8%)
COPD	40 (3.5%)
Depression	62 (5.5%)
Rheumatoid arthritis	31 (3.0%
Fibromyalgia	(20 (1.8%)
Stroke	6 (0.5%)
Hypertension	211 (18.7%)
Migraine headaches	59 (5.2%)
Any rheumatic condition	190 (17%)
Any chronic condition	491 (43.5%)
T-score^a^, mean (SD) min–max	50.62 (17.54) 24.62–89.58
SF-36 Physical functioning, Mean (SD) min–max	83.06 (25.28) 0–100

*COPD* chronic obstructive pulmonary disease; *SF-36* 36-Item Short-form Health Survey; T-score is a item bank-derived physical function estimate, transformed to a scale where mean = 50 (SD = 10), with higher values indicating better function)

^a^According to UNESCO International standard classification of education

### Calibration study participants and data collection procedures

The item pool was administered to 1128 adults enlisted in the Longitudinal Internet Studies for the Social sciences (LISS) panel, an academic research panel administered by Tilburg University in collaboration with Statistics Netherlands, for which a true probability sample was drawn from the Dutch population registers [45]. The LISS panel has been shown to approximate national statistics on the total non-incarcerated population in the Netherlands better than 18 online panels based on non-probability, self-selected samples in terms of coverage of sociodemographic characteristics [46]. Representativeness of the panel has also been demonstrated to approximate a major national face-to-face survey (the Dutch parliamentary election study) conducted by Statistics Netherlands on all tested variables except with respect to coverage of the elderly (>70 years old) and the non-internet population [47]. The module that respondents completed for our study contained the item pool of 72 items, basic demographic information, and the SF-36 physical functioning scale (PF-10), which contains 10 items measuring perceived current limitations in a variety of physical activities on a 3-point response scale. Scores are summed and linearly transformed to range between 0 and 100, with higher scores indicating better functioning. Previous studies have shown the PF-10 to have favorable measurement properties in a variety of settings [48].

Respondents were also asked if they had ever been diagnosed by a physician with any of the following chronic conditions that are prevalent in The Netherlands according to the Dutch National Institute for Public Health and the Environment (www.volksgezondheidenzorg.info): osteoarthritis, diabetes, asthma, chronic obstructive pulmonary disease, depression, rheumatoid arthritis, fibromyalgia, hypertension, stroke, congenital heart disease, or migraine. Finally, respondents were asked to rate the level of difficulty they had filling out the questionnaire and the clarity of the survey in general, including the PF-10 and the other items using 5-point Likert scales with response options ranging from not at all difficult/unclear to extremely difficult/clear.

### Assessment of item quality and IRT assumptions

IRT models for ordered polytomous data assume that the score on a questionnaire item is explained by a (set of) latent trait(s) and the relationship between the observed score and the trait(s) can be described by a monotonically increasing function [16]. The dimensionality of the item pool and the assumption that the expected scores of individual items are continuously non-decreasing across the physical function continuum were examined preceding the IRT analysis. Since all items were newly developed for this study, we used exploratory factor analysis (EFA) with robust weighted least squares estimation on polychoric correlations in Mplus 7.11 to explore the latent structure of the item pool. The number of factors to retain was decided after examining the eigenvalues (i.e., the variances of the extracted factors), and model-data fit was assessed using proposed cut-off values for the fit indices provided by Mplus [49]. Based on the results of this analysis, we decided if a unidimensional or multidimensional IRT model would be used. We excluded items from EFA for which individual response options were selected by <20 respondents since it would be difficult to obtain good estimates of the IRT intersection parameters (described below) at a later stage. Items for which >80% of responses were in either extreme end of the 5-point response scale were also excluded.

The assumption that expected item scores are monotonically increasing throughout the range of trait values was examined using the check for monotonicity function in the Mokken R-package [50]. In this procedure, respondents are grouped based on their rest score (i.e., total score on all items minus the item under consideration) and it is examined if the percentages of respondents endorsing each of an item’s response options are continuously non-decreasing for all levels of the restscore. For each item, the number of violations of monotonicity was counted, as well as the number of statistically significant violations. In case of significant violations, the ‘crit’ statistic quantifies the magnitude of violation. Values >40 are considered to be problematic [51].

### IRT modeling

We considered a series of generalizations of the partial credit model (PCM), which is a Rasch-type model for ordered polytomous data in which the item and person characteristics are located on the same logit scale. The PCM specifies that for an item *i* with *k* = 1.. *m* score categories, the log odds of a score in category *k* instead of *k*−1 by a person *n* depend only on the distance on the measurement continuum between that respondent’s physical function level $\theta_{n}$ and a parameter $\beta_{ik}$ that reflects the location on the scale where a response in both categories is equally likely:

$${\text{LN}}\frac{{P_{nik} }}{{P_{ni(k - 1)} }} = \theta_{n} - \beta_{ik}.$$

The PCM can be generalized by introducing a discrimination parameter that allows the amount of change in the log odds for one unit of change in $\theta_{n}$ to be different between items [52]. If needed, this model can be further generalized to accommodate multiple dimensions. Since these models are nested, we compared their relative fit using a likelihood ratio test and we used an item-level Lagrange multiplier (LM) test [53].

After a model was chosen, we examined the presence of age, educational level, and sex-related differential item functioning (DIF) using LM statistics and associated effect size (ES) statistics, calculated as the expected value of the residuals across three score level groups for item fit and across sociodemographic subgroups considered in the DIF analyses. The residuals were divided by the maximum attainable item score, such that an ES of, for example, 0.10 indicates that the observed average score was 10% different from its expectation under the model [54]. For age, the sample was split into three groups (0–39, *n* = 354; 40–59, *n* = 364; 60+, *n* = 410) and for educational level respondents were classified in three groups in accordance with the International Standard Classification of Education [55]. Pronounced DIF was defined as a *p* value for the LM test <0.01 in combination with an ES of >5%. The IRT models we considered all assume that the item responses depend only on the value(s) of the latent trait(s) in the model. Local stochastic independence was evaluated using Yen’s *Q*3 statistic (i.e., the correlation between fit residuals) [56]. Items with an absolute *Q*3 > ± 0.20 were flagged for possible local stochastic dependence [17, 56, 57]. The impact of local dependence was examined by examining the change in parameter estimates after omitting locally dependent items. All IRT analyses were performed using the MIRT package [58].

### Analysis of measurement properties

A preliminary evaluation of the performance of item bank-derived measures was performed by using the responses to the calibrated items as input for an empirical CAT simulation. The first item to be administered was selected to maximize information at the mean of the distribution of trait levels. The maximum posterior weighted information criterion [59] was used for subsequent item selection, using the expected a posteriori procedure for interim latent score estimation. A standard normal prior was used for both interim item selection and interim score estimation. Final estimates and their standard errors were obtained using the maximum likelihood (ML) method.

#### Measurement precision

The measurement precision of the CAT algorithm with various numbers of administered items was compared with that of the PF-10 and administration of 10 random items from the item bank by plotting the standard errors of the ML estimates of the different measures across the various CAT score levels. We also compared floor and ceiling effects for all administered items, defined as the percentage of persons that selected the highest and lowest response option, respectively. We examined floor and ceiling effects in the total study population as well as in the subpopulation with ≥1 self-reported chronic condition and in the subpopulation with any self-reported rheumatic condition (i.e., rheumatoid arthritis, osteoarthritis, or fibromyalgia).

#### Sensitivity

We compared the ability of the instruments to differentiate between respondents with self-reported osteoarthritis, rheumatoid arthritis, or fibromyalgia (i.e., with a rheumatic condition) versus those without any rheumatic condition. We also compared scores between respondents who self-reported to have been diagnosed with 0, 1, or >1 chronic conditions and respondents in different age groups. We hypothesized that all measures would discriminate between these groups and that T-scores and PF-10 scores would decrease with the number of chronic conditions and age. To compare discriminative ability of the different instruments, relative efficiency coefficients were obtained [60].

## Results

### Sample characteristics

Some characteristics of the sample are summarized in Table 1. Physical function scores were high on average, but respondents of all functional levels assessed by the PF-10 were represented in the sample.

### IRT assumptions

Twenty-five items with ≥80% of responses in the “with no difficulties” category were excluded. All remaining items had >20 responses in each response category. EFA on the remaining 47 items revealed three factors with eigenvalues >1 and a strong first factor, with a first-to-second eigenvalue ratio of 13.14. The first three eigenvalues were 34.95, 2.66, and 1.38 and the first factor explained 77% of the total variance. Furthermore, all items had significant (*p* < 0.05) loadings on the first factor, generally in excess of 0.77, except for item 2 (sewing clothes by hand using needle and thread), for which the loading on the first factor was 0.53. Except for RMSEA (0.09), all fit indices suggested a good fit (TLI = 0.96, CFI = 0.96), SRMSR = 0.10 and did not improve much for the two-factor solution, further supporting a unidimensional measurement model. For the one-factor model, only 1% of item pairs had residual correlations >0.20. Based on these results, we concluded that a unidimensional item response model was probably most suitable for these data. In the Mokken analysis, six items were identified with each one violation of monotonicity. However, none of these violations were statistically significant and the ‘crit’ statistic was <40 in all cases (Max *crit* = 7). All items had scalability coefficients >0.30, indicating that respondents can be ordered on the latent continuum using the total score (Table 2).

**Table 2:** Item characteristics of the final 41 items of the Rasch Everyday Activities Limitations item bank

ICF code	Item	$\beta_{1}$	$\beta_{2}$	$\beta_{3}$	$\beta_{4}$	Effect size (%)	*H*_*ij*_ coefficient
d65506	Cleaning the oven	29	13	9	0	0.2	0.68
d6201	Clothes shopping	27	21	10	0	0.4	0.69
d65502	Turning in light bulbs overhead	29	21	15	6	0.2	0.67
d65501	Sweeping outside surfaces	31	20	18	3	0.6	0.67
d6102	Moving light furniture	36	25	12	0	0.2	0.70
d6201	Carrying groceries	35	20	16	8	0.8	0.74
d6402	Cleaning bathroom	33	23	18	7	0.8	0.73
d6402	Making a double bed	37	23	17	5	0.2	0.71
d65502	Tightening nuts and bolts	29	25	18	12	0.4	0.67
d6402	Cleaning windows	32	22	17	14	0.6	0.73
d6600	Helping children dress	30	19	14	23	0.8	0.67
d65505	Walking a leashed dog	24	21	16	27	0.6	0.67
d6600	Helping children wash and dry	30	22	10	26	0.8	0.67
d6503	Washing the car	33	26	20	14	1.6	0.73
d6403	Vacuuming stairs	36	23	19	17	1.4	0.75
d6550	Heavy ironing	34	27	17	17	1.2	0.58
d6201	Carrying bags of fruit	41	22	20	16	1.2	0.76
d6601	Pushing a wheelchair 15 min	37	27	21	18	0.8	0.75
d6601	Helping others up from a seating position	39	29	19	18	0.8	0.74
d65501	Weeding the front yard	43	30	20	16	1.4	0.70
d6405	Depositing two full trash bags	43	26	23	20	1.2	0.75
d65505	Trimming grass using scissors	46	35	26	21	1.4	0.74
d6601	Helping others up from a laying position	50	34	26	21	0.8	0.74
d65505	Pruning hedges	47	36	29	25	1.2	0.73
d65505	Mowing the lawn with a hand mower	45	39	32	25	1.6	0.74
d6102	Redecorating the living room	57	41	32	21	1.2	0.75
d6601	Helping adults in an out of a tub bath	54	40	30	29	0.4	0.72
d65501	Repairing carpentry around the house	47	40	32	36	0.4	0.68
d65501	Painting living room walls	50	40	33	32	2.0	0.72
d65501	Making repairs around the house	52	40	31	32	0.0	0.67
d6102	Assembling a bookcase or wardrobe	53	38	34	30	0.6	0.73
d65505	Spading the garden	57	43	39	31	2.6	0.77
d65501	Hanging wallpaper	54	43	35	40	1.2	0.68
d6102	Carrying moving boxes inside the home	62	44	35	31	1.0	0.75
d65501	Painting the living room	59	42	37	35	2.6	0.74
d6601	Helping a disabled person up two stairs	68	46	37	30	1.0	0.72
d65505	Doing yard work on knees for 1 h	63	48	39	33	2.0	0.75
d6102	Moving heavy furniture	71	49	40	33	0.8	0.75
d6102	Helping carry upstairs a washing machine	70	59	53	43	0.8	0.78
d6102	Helping carry inside heavy furniture	77	59	49	39	1.0	0.78
d6102	Helping someone carry upstairs a sofa	76	61	47	41	1.0	0.78

*ICF* International Classification of Functioning, Disability and Health; *β* Partial Credit Model Category intersection parameter; *Effect size* item fit statistic reflecting mean residuals across three score level groups; *H-coefficient Loevinger’s* coefficient of scalability, values >0.3 are considered .3 ≤ *H*_*ij*_ < 0.4 indicates useful but weak *scalability*. 0.4 ≤ *H*_*ij*_ < 0.5 indicates medium *scalability*. *H*_*ij*_ > 0.5 indicates excellent scalability

### IRT model fitting

In an initial evaluation of all 46 items, the LR test yielded a value of *χ* ^2^ = 646,826, df = 46, *p* < 0.01, indicating superior fit for the GPCM compared with PCM. However, for both models, ESs were generally of small magnitude (i.e., ES <2%) and the root mean squared difference ES was <0.6%, suggesting that both models performed similarly in terms of reproducing the item response data. Only item 2 met the criteria for substantial lack of fit in the PCM calibration (LM = 107.39 *p* < 0.01, ES = 5.8%), but not the GPCM calibration. This was likely due to its weak relation with the general factor observed earlier in the EFA.

Although GPCM performed better according to the LR test, we elected to delete item 2 and proceed with the more parsimonious PCM. Inspection of the matrix with *Q*3 statistics revealed that 6% of the item pairs had an absolute *Q*3 >0.20. After removing the item with the highest average *Q*3, item threshold parameters of the remaining items changed by a maximum of 0.13 and the mean absolute difference between threshold parameters in the original calibration and the re-estimated item parameters was 0.04 (SD = 0.03). After 10 items were removed, the mean absolute difference was 0.04 (SD = 0.01), suggesting a small impact of local dependence on the parameter estimates. For age or educational attainment, none of the items had substantial DIF in the PCM calibration. However, for 2 items, involving preparing meals and washing clothes by hand, scores were >5% lower for male respondents than expected based on their overall level of function, while 3 items all involving repairing vehicles were more difficult for female respondents.

A summary of item characteristics of the final item bank with 41 items is presented in Table 2, organized from least to most difficult to perform activity. The derived IRT theta scores of the final item bank were transformed into T-scores with a mean of 50 and standard deviation of 10. A T-score of 50 corresponds to the mean level of physical function in the sample and higher scores indicate better function. It can be seen in the table that most of the intersection parameters are localized below the mean, indicating that the items discriminate best between lower levels of physical function. However, the item bank also includes some activities that are well suited to assess respondents with high levels of function (bottom of Table 2).

### CAT measurement properties

**Fig. 1:**
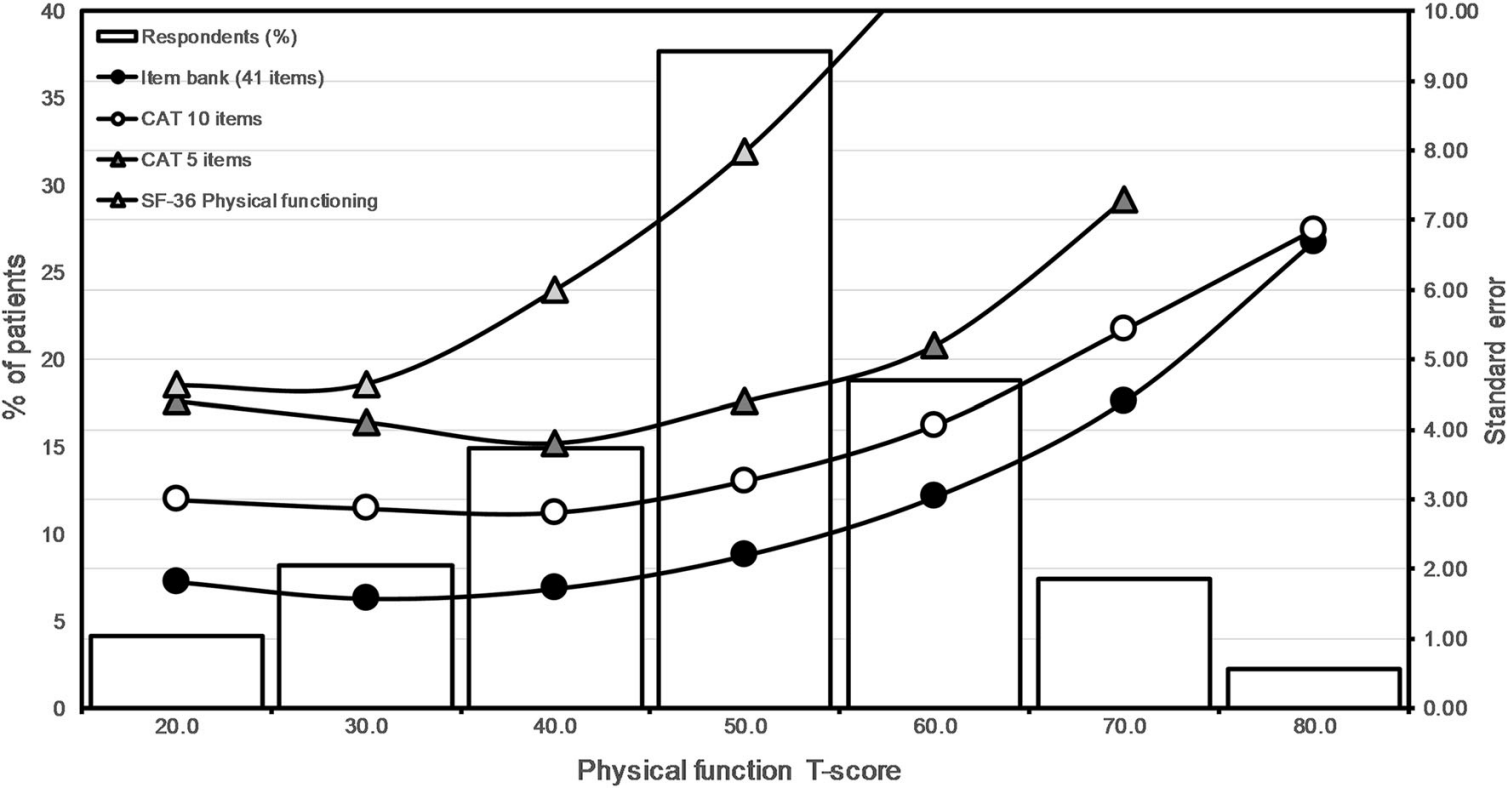
Measurement precision of a simulated CAT with 5 and 10 items, compared with the PF-10 and the entire item bank

The correlation between the physical function estimates obtained using the algorithm with 5 items and the estimates for the full item bank obtained in the statistical software used to estimate the item parameters (MIRT) was 0.92 (Table 3) and increased further with the number of administered items. The CAT scores ranged from 22.05 to 79.24. Figure 1 shows that measurement precision of CAT exceeded that of the PF-10, even with only 5 items. Measurement precision was high across the measurement continuum for the CAT with 10 items (SE <7, which roughly corresponds to a classical reliability coefficient of 0.90). By contrast, for the PF-10 this level of precision was observed only for respondents with below average T-scores.

**Table 3:** Empirical CAT simulation

	T-score, mean (SD)	SEM, mean (SD)	Correlation with full item bank	Floor (%)	Ceiling (%)
Random-10 items	51.89 (14.90)	5.50 (1.80)	0.92	0.4	18.7
CAT-5 items	52.10 (14.92)	5.50 (1.80)	0.92	0.7	15.6
CAT-10 items	54.27 (17.14)	4.00 (1.20)	0.96	0.3	13.1
CAT-15 items	54.43 (17.77	3.60 (130)	0.94	0.1	12.8
PF-10				2.3	38.1

*Random-10* administration of 10 random item bank items; *CAT* computerized adaptive test; *PF-10* 36-Item Short-form Health Survey, physical functioning scale; *SEM* standard error of measurement

9.4% of respondents had a T-score that exceeded 80 based on the administration of all items. Inspection of response patterns indicated that these respondents scored 0 on all 41 items. These respondents were more frequently male (66.9% vs. 43.7%, *p* < 0.01), significantly younger (*M*_s0_ = 38.77, *M*_s>0_ = 50.29, *p* < 0.01) and significantly less likely to have any chronic (12.8% vs. 44.1%, *p* < 0.01) or rheumatic (1.5% vs. 18.9%, *p* < 0.01) condition than respondents with scores > 0. In terms of ceiling and floor effects, the CAT with 5 items outperformed both the PF-10 and the random administration of 10 REAL items (Table 3). Ceiling effects for the CAT-10 were almost completely resolved for respondents with any rheumatic condition (*n* = 2, 1.1%) and the subpopulation of respondents with any medical condition (*n* = 26, 5.3%). The CAT with 5 items performed equally well as 10 random items in terms of ceiling effects and measurement precision, clearly highlighting the added value of the computerized statistical optimization approach.

#### Sensitivity

Both CAT-10 and PF-10 discriminated between respondents as indicated by significant F-Tests. T-scores and PF-10 scores increased with the number of chronic conditions and age, and respondents with rheumatic conditions had higher scores than those who did not (Table 4). The relative efficiency of the CAT-10 was greatly superior to the PF-10 for all comparisons.

**Table 4:** Discriminative ability

	Age (years) Mean (SD)			*F* (RE)	Rheumatic condition Mean (SD)		*F* (RE)	Number of conditions Mean (SD)			*F* (RE)
	0–39 (*n* = 354)	40–59 (*n* = 362)	60+ (*n* = 410)		No (*n* = 932)	Yes (*n* = 190)		0 (*n* = 631)	1 (*n* = 309)	2+ (*n* = 178)	
PF-10	90.54 (22.07)	85.42 (24.28)	74.59 (26.30)	42.83 (1.00)	86.35 (24.05)	66.94 (25.08)	101.22 (1.00)	88.18 (23.55)	84.82 (20.50)	61.76 (27.85)	88.89 (1.00)
CAT-10	63.67 (13.47)	57.29 (15.25)	43.75 (15.99)	173.31 (4.12)	57.51 (16.04)	38.61 (13.57)	231.09 (2.20)	59.80 (15.35)	52.16 (15.69)	38.49 (14.92)	137.63 (1.50)

*CAT-10* computerized adaptive test with 10 items; *SF-35 PF10* 36-Item Short-form Health Survey, physical functioning scale; *RE* relative efficiency coefficient

#### Respondent feedback

Respondents generally found the questions easy to answer (5-point Likert Mean = 1.5, SD = 1.0) and the wording clear (5-point Likert mean = 4.3, SD = 1.0). 70 patients who found the questions either unclear (n = 83, 7.3%) or difficult to answer (n = 80, 7.1%) motivated their response. 23 respondents reported that a ‘non-applicable’ response option would have been useful, 11 respondents found some of the questions difficult to answer because they had no experience in doing some of the activities, 5 further respondents directly attributed this difficulty to their gender roles. 7 respondents found the response options difficult to use. The remaining comments pertained to other parts of the survey (e.g., the PF-10 or the question about chronic conditions).

## Discussion

In this paper we report on the development, calibration, and initial evaluation of REAL, a new item bank for measuring complex activities of daily living. The results demonstrate that the items fulfill the strong requirements imposed by the Rasch-type IRT models and support the psychometric quality of the item bank. Moreover, we were able to demonstrate superior measurement performance of CAT versus a traditional pen and paper questionnaire, the PF-10, with particular benefits in higher regions of function.

The current version of the REAL item bank has 41 items that cover most of the second-level codes of the ICF domestic life chapter and were in most cases derived directly from patient responses, which supports the content validity of the item bank [61]. As hypothesized, reliable scores were seen across a wider range of score levels for a CAT with 5 items compared with the PF-10. Although the ceiling effects present in the PF-10 could be reduced by a factor of 3 using CAT with 10 items, some respondents still obtained the highest possible score. In principle, the range of scores that can be reliably measured could be extended further by including even more extreme items (e.g., running 16 miles). However, respondents at the ceiling were typically healthy, younger men, while only slight ceiling effects were observed in the subpopulation with at least one self-reported chronic condition. This suggests that the item bank should yield reliable scores with low ceiling effects in most clinical populations for which physical function levels are likely to be lower than in the subpopulation of respondents with a self-reported chronic condition in the sample studied here.

Although these findings are encouraging, a potential concern with the type of activities included in the item bank is that the frequency with which respondents engage in individual activities may vary with factors such as gender or age. Perceived skill and experience with an activity could therefore contribute to item response behavior and undermine the validity of the scores [37]. However, we used quite conservative definitions of unacceptable DIF and found that no items functioned differently by education or age, while we removed the 10% of evaluated items that were responded to differently by male or female respondents. Since the presence of DIF of the magnitude we tolerated has been shown to have negligible impact on total scores in previous studies, the results of the present study suggest that the validity of scores obtained using the item bank should not be affected much by differential item functioning due to age, sex, or educational level.

The item bank was calibrated using the Rasch-based PCM which is an IRT model that implies that all the information about a person’s trait level is provided by the unweighted item scores [62]. The finding that the PCM described the response data well therefore supports the validity of a scoring rule based on summing or averaging of individual items, in cases where respondents are compared using the same set of items (e.g., using a short-form derived from the items in the item bank). Since the patterns of item responses contribute no information to trait level estimates they can also be ignored in other conceivable applications of the item bank such as when developing a crosswalk between a short-form and the T-score metric.

A strength of the study is that we were able to center the scale using a representative sample of the Dutch general population without having to resort to oversampling of respondents with chronic conditions, which would have undermined the validity of norms and might have distorted the item parameters [63]. The well-documented representativeness of the LISS panel as well as the finding that the mean PF-10 score approximates the published Dutch national population norms for this scale [64] supports the use of item bank-derived T-scores for comparing physical function levels to the Dutch adult population. Since items and persons are located on the same scale, a different way to interpret the scores of individuals is in reference to the item steps that are ‘dominated’ by a person and vice versa. For example, a person with a T-score of 50 has a higher than 50% change to report being able to perform each of the first 25 items of Table 2 without any difficulty.

A limitation of the design of the study is that sparse information was available to explore construct validity in detail and no information was available to study responsiveness. Moreover, the performance of the adaptive testing algorithm was studied using an empirical simulation. Since these item responses were also used to calibrate the item bank, results presented here should be replicated in an actual CAT administration. Work is currently underway to assess the item bank and operational CAT in various rheumatic diseases. An important part of that work will be to investigate the extent to which the item response models obtained here can also be used in populations with different types of disease-related disability and to examine the need for extending the range of the scale to be able to reliably assess respondents with more severe levels of disability than generally observed in the present study. If needed, this could be achieved in several ways. Some of the items that proved to be too easy for respondents in the present study might be calibrated at a later stage. Alternatively, some of the many ADL items that are available in currently validated questionnaires can be included in the item bank.

In the present study, we have introduced a new item bank focusing on complex activities of daily living that we hope will prove useful for assessing physical function in various populations.

## Electronic supplementary material

Below is the link to the electronic supplementary material.

Supplementary material 1 (DOCX 14 kb)
