# Supplementary material for: Measuring everyday functional competence using the Rasch assessment of everyday activity limitations (REAL) item bank
Source: Qual Life Res. 2017 Jun 21;26(11):2949–59. doi: 10.1007/s11136-017-1627-0 (PMC5655561; doi:10.1007/s11136-017-1627-0)
Supplement: Supplementary file 1 — Supplementary material 1 (DOCX 14 kb) [file 11136_2017_1627_MOESM1_ESM.docx]

Supplementary Table 1. Characteristics of convenience sample

|  | RA  (n=30) | Gout  (n=31) | FM  (n=17) | OA  (n=25) |
| --- | --- | --- | --- | --- |
| Age, years | 59.5 (11.4) | 59.9 (8.7) | 42.4 (13.2) | 59.1 (9.8) |
| Time since diagnosis, yrs. | 4.8 (4.7) | 2.6 (3.5) | 3.1 (5.7) | 3.8 (4.1) |
| Sex, n (%)female | 76%) | 16% | 82% | 80% |
| Educational level*, n (%) |  |  |  |  |
| Low | 10 (34.5%) | 14 (45.2%) | 6 (35.3%) | 8 (32.0%) |
| Middle | 16 (55.2%) | 12 (38.7%) | 10 (58.8%) | 14 (56.0%) |
| High | 3 (10.3%) | 5 (16.1%) | 1 (5.9%) | 3 (12.0%) |
| Occupational status, n (%) |  |  |  |  |
| Remuneratively employed | 10 (33.3%) | 17 (54.8%) | 10 (58.8%) | 11 (45.8%) |
| Housekeeper | 6 (20.0%) | 1 (3.2%) | 0 (0.0%) | 6 (25.0%) |
| Student | 1 (3.3%) | 0 (0.0%) | 2 (11.8%) | 0 (0.0%) |
| Unemployed | 2 (6.6%) | 2 (6.5%) | 2 (11.8%) | 0 (0.0%) |
| Pensioned | 3 (9.9%) | 2 (6.5%) | 2 (11.8%) | 3 (12.5%) |
| Other | 8 (26.7%) | 9 (29.0%) | 1 (5.9%) | 4 (16.7%) |

RA = Rheumatoid arthirits ; FM = fibromyalgia; OA = osteoarthritis; Values are mean (SD) unless reported otherwise. ). * according to UNESCO International standard classification of education.
